# Colonization of plant substrates at hydrothermal vents and cold seeps in the northeast Atlantic and Mediterranean and occurrence of symbiont-related bacteria

**DOI:** 10.3389/fmicb.2015.00162

**Published:** 2015-02-27

**Authors:** Kamil M. Szafranski, Philippe Deschamps, Marina R. Cunha, Sylvie M. Gaudron, Sébastien Duperron

**Affiliations:** ^1^Sorbonne Universités, UPMC Univ. Paris 06, UMR 7208, Adaptation aux Milieux ExtrêmesParis, France; ^2^UMR MNHN UPMC CNRS IRD UCBN 7208, Biologie des Organismes Aquatiques et EcosystèmesParis, France; ^3^UMR8079 Unité d'Ecologie, Systématique et Evolution, CNRS Université Paris-Sud 11Orsay, France; ^4^Departamento de Biologia and CESAM, Universidade de AveiroAveiro, Portugal; ^5^Institut Universitaire de FranceParis, France

**Keywords:** cold seeps, colonization, deep-sea, symbiont, hydrothermal vents, wood falls

## Abstract

Reducing conditions with elevated sulfide and methane concentrations in ecosystems such as hydrothermal vents, cold seeps or organic falls, are suitable for chemosynthetic primary production. Understanding processes driving bacterial diversity, colonization and dispersal is of prime importance for deep-sea microbial ecology. This study provides a detailed characterization of bacterial assemblages colonizing plant-derived substrates using a standardized approach over a geographic area spanning the North-East Atlantic and Mediterranean. Wood and alfalfa substrates in colonization devices were deployed for different periods at 8 deep-sea chemosynthesis-based sites in four distinct geographic areas. Pyrosequencing of a fragment of the 16S rRNA-encoding gene was used to describe bacterial communities. Colonization occurred within the first 14 days. The diversity was higher in samples deployed for more than 289 days. After 289 days, no relation was observed between community richness and deployment duration, suggesting that diversity may have reached saturation sometime in between. Communities in long-term deployments were different, and their composition was mainly influenced by the geographical location where devices were deployed. Numerous sequences related to horizontally-transmitted chemosynthetic symbionts of metazoans were identified. Their potential status as free-living forms of these symbionts was evaluated based on sequence similarity with demonstrated symbionts. Results suggest that some free-living forms of metazoan symbionts or their close relatives, such as Epsilonproteobacteria associated with the shrimp *Rimicaris exoculata*, are efficient colonizers of plant substrates at vents and seeps.

## Introduction

Hydrothermal vents and other chemosynthesis-based ecosystems like cold seeps are deep-sea hotspots of primary production, which is ensured by chemoautotrophic prokaryotes, many of which live in symbiosis with various metazoans (Van Dover, 2000; Dubilier et al., 2008). The deep-sea ecosystem can also benefit from significant input of exogenous sources of carbon such as plant remains of different origins, like sunken wood (Wolff, 1979). Wood falls contain cellulose, hemicellulose and lignin, i.e., energy-rich polysaccharides that can be degraded by microbial assemblages. This results in the production of reduced compounds such as hydrogen sulfide, dihydrogen and methane, providing a niche for thiotrophic and methanotrophic bacteria (Leschine, 1995; Palacios et al., 2006). Wood falls, but also whale skeletons and others large inputs of organic material, are quickly localized and efficiently colonized by opportunistic fauna (Turner, 1973; Bennett et al., 1994; Baco and Smith, 2003; Smith and Baco, 2003; Laurent et al., 2013). Reducing conditions with elevated sulfide concentrations around degrading organic falls also constitute a prime environment for metazoans with sulfide-oxidizing symbioses (Duperron et al., 2008; Lorion et al., 2009; reviewed in: Dubilier et al., 2008; Laurent et al., 2009; Treude et al., 2009). Seep and vent ecosystems are distributed worldwide but are often separated by large distances, and various authors postulated that wood and whale falls could act as stepping stones between habitats (Vrijenhoek, 2010). Nevertheless, the most successful animal colonizers remain specialists for one substrate type, remarkable examples including *Xylophaga* spp. or *Xyloredo* spp. bivalves on wood (Turner, 1973; Distel and Roberts, 1997) and *Osedax* spp. polychaetes on bones (Rouse et al., 2004; Glover et al., 2005).

Despite the significance of sunken plant substrates as locale organic enrichments, studies on the associated microbial assemblages remain scarce. The first trial to investigate and compare free-living and attached prokaryotic assemblages from sunken wood at several sites and water depths was carried out by Palacios et al. (2009). Authors combined electron microscopy and molecular fingerprinting (capillary electrophoresis single-stranded conformation polymorphisms—CE-SSCP) to test the influence of depth of immersion, geographic location, deployment time, and wood type on the structure of microbial assemblages (Palacios et al., 2009). The phylogeny and diversity of Bacteria and Archaea associated with wood falls was recently investigated using clone libraries (Fagervold et al., 2012), and authors demonstrated the occurrence of various free-living sulfate-reducing and sulfur-oxidizing bacteria (SRB and SOX respectively) and methanogenic archaea. This microbial community was modified with time of immersion because of changes in wood chemistry. The combination of ARISA fingerprinting and 454 pyrosequencing recently allowed Bienhold et al. (2013) to gain a more detailed overview of bacterial assemblages in a pine wood deployment lasting 1 year and surrounding sediments at a cold seep site in the Eastern Mediterranean Sea. They established that wood-boring bivalves, cellulolytic and sulfate-reducing bacteria colonized the organic substrate first, and then attracted chemosymbiotic fauna (Bienhold et al., 2013). Finally, using pyrosequencing, Fagervold et al. (2013) also demonstrated that the microbial diversity in oak cubes deployed at the Blanes Canyon (Western Mediterranean Sea) was significantly higher compared to cubes deployed in the adjacent open slope (Fagervold et al., 2013). To date, these high-throughput diversity studies include a single region, use only one substrate type, and are all based on different experimental designs, and as a consequence knowledge of deep-sea wood-associated microbial assemblages is still scarce.

Organic fall-associated and vent/seep faunas include many metazoans harboring chemosynthetic symbionts, and many of these symbionts are environmentally acquired anew at each generation (Gros et al., 1996; McFall-Ngai, 2000; Won et al., 2003; Nussbaumer et al., 2006; Duperron et al., 2008; Lorion et al., 2009; reviewed in: Dubilier et al., 2008; Bright and Bulgheresi, 2010; Vrijenhoek, 2010). This mode of symbiont transmission implies the existence of free-living forms of symbionts which may infect the appropriate host, and for which symbiotic lifestyle may be facultative (Bright and Bulgheresi, 2010). Only few studies successfully documented the occurrence of such free-living symbionts in marine shallow waters (Lee and Ruby, 1992; Gros et al., 2003; Aida et al., 2008), even less in the deep-sea (Harmer et al., 2008). In which habitat and numbers do these free-living forms exist thus remains an open question.

Since 2006, colonization devices (CHEMECOLIs) filled with wood cubes and alfalfa grass have been deployed for various periods of time at several chemosynthesis-based sites located in the Eastern Mediterranean, the Gulf of Cadiz, the Mid-Atlantic Ridge and the Haakon Mosby Mud Volcano (Gaudron et al., 2010; Cunha et al., 2013). In this study, we characterize the bacterial communities colonizing these organic substrates. The aims are to identify bacteria colonizing the substrates, to evaluate the effect of region, type of substrate, depth, duration of deployment, and temperature on the bacterial assemblages, and to screen for potential free-living forms or relatives of metazoan-associated chemosynthetic symbionts. For this, a 454 pyrosequencing-based approach on a 16S rRNA-encoding gene fragment is employed.

## Materials and methods

### Sampling

**Figure 1:**
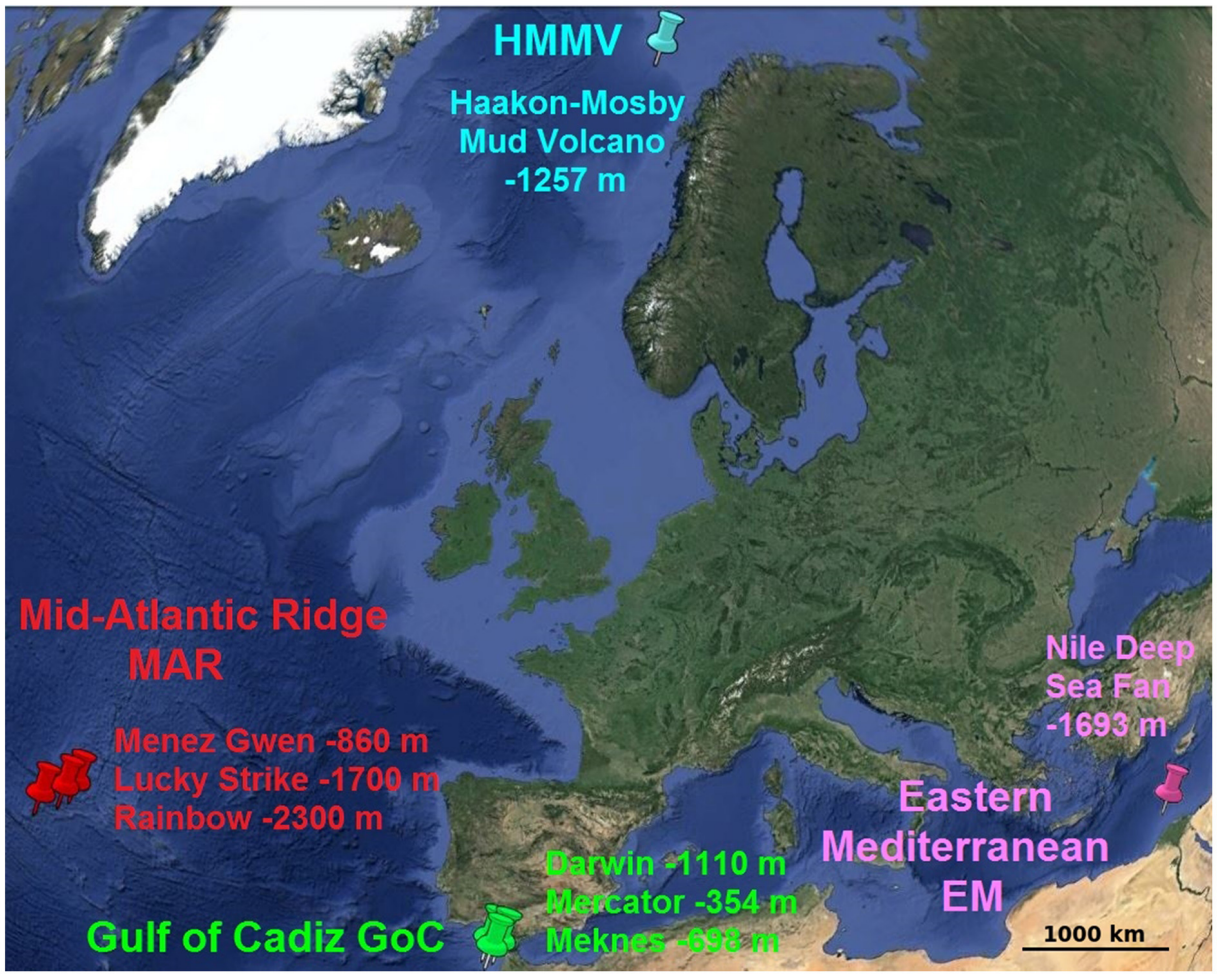
**Map of sampling sites and their depths, located in 4 geographical regions**.

Thirteen sets of standardized colonization devices (CHEMECOLI: CHEMosynthetic Ecosystem COlonization by Larval Invertebrates, described by Gaudron et al., 2010) were deployed at 8 hydrothermal vents and cold seeps sites located in 4 distinct geographic areas (Figure 1): the Mid-Atlantic Ridge (MAR, 3 sites), the Gulf of Cadiz (GoC, 3 sites), the Eastern Mediterranean (EM, 1 site), and the Norwegian Sea (Haakon Mosby Mud Volcano, HMMV, 1 site). Each set consisted of two CHEMECOLIs, one filled with dried alfalfa grass (A) and the second with 2 cm pine wood cubes (W). Deployment sites were located at depths ranging from 354 to 2300 m. CHEMECOLIs were deployed a few meters away from any visible fluid escape and recovered in individual sterile-water filled hermetic boxes after periods of 10 days to 3 years. Operations were performed by various ROVs (Remotely Operated Vehicles) over the course of 13 cruises spanning over years 2006–2013 (Table 1). Immediately after recovery, pieces of alfalfa and wood cubes were randomly selected in a cold room, fixed in 96% ethanol and stored at 4°C until DNA extraction. Substrates from a short-term deployment at Menez Gwen were also frozen in liquid nitrogen and stored at −80°C in order to compare different sample fixation procedures (MAR-A-V-C, MAR-W-V-C). Non-deployed substrates were used as controls (Contr-A and Contr-W).

**Table 1:** **Main characteristics of samples used for sequence analyses**.

**Sample name**		**Region**	**Site**	**Depth**	**Days**	**Temperature**	**Site localization**		**Deployment date**	**Cruise**	**Recovery date**	**Cruise**
									****		****	
**Alfalfa**	**Wood**											
**Contr-A**	-	**Negative control**										
GoC-A-I	GoC-W-I	Gulf of Cadiz	Mercator	354 m	289	12	35°17.916′N	6°38.709′W	19/05/2007	JC10	03/03/2008	64PE284
GoC-A-II	GoC-W-II	Gulf of Cadiz	Mercator	354 m	731	12	35°17.916′N	6°38.709′W	19/05/2007	JC10	19/05/2009	Belgica cruise ST0914
GoC-A-III	GoC-W-III	Gulf of Cadiz	Meknes	698 m	445	11	34°59.09′N	7°04.42′W	01/03/2008	64PE284	20/05/2009	Belgica cruise ST0914
GoC-A-IV	GoC-W-IV	Gulf of Cadiz	Darwin	1110 m	729	10	35°23.523′N	7°11.513′W	21/05/2007	JC10	19/05/2009	Belgica cruise ST0914
EM-A-I	EM-W-I	Eeastern Mediterranean	NDSF	1693 m	357	13	32° 31.9772′N	30° 21.1779′E	18/11/2006	Bionil	10/11/2007	Medeco
MAR-A-I	MAR-W-I	Mid-Atlantic Ridge	Rainbow	2300 m	328	3.7	36° 13.766′N	33° 54.192′W	21/08/2006	Momareto	15/07/2007	Momardream
MAR-A-II	MAR-W-II	Mid-Atlantic Ridge	Rainbow	2279 m	414	3.7	36°13.7454′N	33°54.0513′W	14/07/2007	Momardream	31/08/2008	MOMAR08
MAR-A-III	MAR-W-III	Mid-Atlantic Ridge	Lucky Strike	1696 m	1112	4.6	37°17.339′N	32°16.533′W	04/09/2006	Momareto	20/09/2009	Bathyluck
HMMV-A-I	HMMV-W-I	Haakon-Mosby Mud Volcano	HMMV	1257 m	388	2	72°00.1443′N	14°43.2282′E	06/06/2006	Vicking	29/06/2007	ARK XXII/1b
HMMV-A-II	HMMV-W-II	Haakon-Mosby Mud Volcano	HMMV	1257 m	752	2	72°00.3390′N	14°43.2209′E	03/07/2007	ARK XXII/1b	24/07/2009	ARKXXIV/2
MAR-A-IV	MAR-W-IV	Mid-Atlantic Ridge	Rainbow	2275 m	10	3.7	36°13.7553′N	33°54.11′W	31/08/2008	MOMAR08	10/09/2008	MOMAR08
-	EM-W-II	Eeastern Mediterranean	NDSF	1697 m	14	13	32°31.993′N	30°21.193′E	04/11/2006	Bionil	18/11/2006	Bionil
MAR-A-V	MAR-W-V	Mid-Atlantic Ridge	Menez Gwen	860 m	13	4	37°50.6786′N	31°31.1493′W	05/08/2013	BioBaz	18/08/2013	BioBaz

*Environmental data for each site were used in redundancy analysis (RDA). Sample nomenclature is as follows: region-substrate-deployment reference. Sites are: Contr, control; GoC, Gulf of Cadiz; EM, eastern Mediterranean; MAR, mid-Atlantic Ridge; HMMV, Haakon Mosby mud volcano. Substrate: alfalfa (A) or wood (W). Deployment reference from I to V*.

### DNA extraction and 454 pyrosequencing

Samples were screened under a dissecting microscope to check for the absence of animal material (tissue, eggs, and larvae). Small pieces (2–3 g) of wood cubes were cut in thin slices and homogenized (3 × 30 s) in a metal jar with beads (12 mm diameter) using a Retch MM301 BeadBeater (Retch, Germany). Approximately equal volume of alfalfa samples were ground to powder in liquid nitrogen. A modified Doyle and Doyle (1987) DNA extraction protocol was applied. About 200–300 mg of powder was mixed with 1 mL of preheated CTAB isolation buffer containing 2% hexadecyltrimethylammonium bromide, 1.4 M NaCl, 0.2% β-mercaptoethanol, 20 mM EDTA, 100 mM Tris-HCl, 1% PVP, and 0.2 mg × mL-1 proteinase K. Samples were agitated (1 h, 60°C) then mixed with one volume of chloroform-isoamyl alcohol (24:1). Nucleic acids from the upper aqueous phase were precipitated by isopopropanol and centrifuged. Dry DNA pellets were re-suspended in sterile water. Concentration and quality were checked. DNA extraction was performed in duplicate, using another randomly selected wood cube or alfalfa grass sample from the same CHEMECOLI, resulting in two replicates (region-substrate-nb-1 and region-substrate-nb-2).

Primers V5V6_F (CAAACAGGATTAGATACCCTG) and V5V6_R (CGTTRCGGGACTTAACCCAACA) (designed by GENOSCREEN) were used to amplify the V5-V6 hypervariable region corresponding to regions 770–1094 in the *E.coli* 16S rRNA-encoding gene. Short (~300 bp) pyrotags were sequenced by 454 pyrosequencing (GsFLX-Roche Diagnostics, GENOSCREEN, France). A 10-bp molecular identifier (MID) tag was inserted between the GS-FLX adapter and the specific primer to facilitate further sequence binning. PCR products were purified and quantified by Picogreen (Invitrogen, USA) and the same amount of amplicons was mixed prior to 454 pyrosequencing. 5/8 of a plate was used to sequence 52 samples (including replicates), expecting a minimum of 7700 reads per sample. Sequences have been submitted to MG-RAST (http://metagenomics.anl.gov/linkin.cgi?project=9917) with MG-RAST ID's ranging from 4571074.3 to 4571125.3 (Table S1).

### Data analyses

Resulting binary “.sff” files were extracted using Mothur (Schloss et al., 2009). Sequences shorter than 250 bp, longer than 350 bp and containing Ns were eliminated from further analysis. Filtered sequences were sorted by their MID sequences into separate Fasta files. Typical 454 sequencing errors and PCR single base errors were screened using the PyroNoise and SeqNoise modules of the AmpliconNoise software (Quince et al., 2011) with default parameters. Sequences with MIDs and primers removed were used to generate a Needleman-Wunsch distance matrix (function NDist) and clustered into operational taxonomic units (OTUs, function Fcluster) using the same software. The matrix of sequence abundances per OTU was generated using Python scripts using 97% identity threshold for OTU definition (Dataset_1 in Table S1). The 10 best hits (maximal *e*-value = 1 e^−10^) for each OTU were found using BLAST (Altschul et al., 1997) using the SSURef_NR99_115 Silva database (Quast et al., 2013), and added to the OTU abundance matrix.

### Analyses of OTU abundances and samples comparison

The microbial α-diversity indices (Shannon and Invsimpson), community microbial richness indices (Chao1 and ACE) and rarefaction data were computed with Mothur on Dataset_1 (Schloss et al., 2009). The second dataset was prepared by eliminating all sequences present in less than 5 copies in the total number of reads per sample (rare OTUs eliminated) and was screened for symbiont-related sequences (Dataset_2 in Table S1). For further community abundance and statistical analyses, OTUs containing in total less than 40 sequences, which represents ~1% of the total number of sequences in the smallest sample (4543) were eliminated, thus generating a reduced data matrix with abundant sequences (Dataset_3 in Table S1). All statistical analyses were performed using R (R Development Core Team, 2013). Non-Metric Multidimensional Scaling (NMDS) was performed using “metaMDS” function in the MASS package. Average agglomerative clustering dendrograms were generated using Unweighted Pair-Group Method with arithmetic Average (UPGMA) on a Bray-Curtis distance matrix. Abundance data were transformed using the Hellinger method (Legendre and Gallagher, 2001) and then used for transformation-based redundancy analyses (tb-RDA). Substrate type and region were used as factors and water temperature, depth, and deployment time were used as variables into the constrained RDA, in order to estimate their contribution to the global variance. Significance was assessed using ANOVA permutation tests.

### Identification of OTUs related to metazoan-associated bacterial symbionts

The Dataset_2 was used for screening for symbiont-related sequences. Sequences from this dataset were blasted (with 97% identity threshold) against a local database (Supplementary File), containing sequences of bacteria living in symbiosis with deep-sea fauna (Potential_symbionts in Table S1). These sequences were then blasted against public database and their blast hits definitions were then filtered by scanning for the “symbiont” key word (Confirmed_symbionts in Table S1). In parallel, a phylogenetic analysis was computed in order to evaluate the phylogenetic relationship of sequences to known symbionts. For this, 16S rRNA sequences of documented symbiotic bacteria from vent and seep metazoans and several best blast hits from our OTUs were aligned with SINA Web Aligner (Pruesse et al., 2012) and truncated to the V5-V6 region. Symbiont-related OTUs from this study were added to this dataset, aligned with ClustalX (Larkin et al., 2007), and alignments were manually checked. Phylogenetic relationships among sequences were estimated from a 250-bp alignment with MEGA6 (Tamura et al., 2013) using distance methods and neighbor-joining. Bootstrap values were computed on 1000 replicates.

## Results

### Preparation of datasets

Bacterial diversity was investigated on 25 individual CHEMECOLIs filled with either pine wood cubes or alfalfa and deployed for periods of 10–1112 days in four areas in the North East Atlantic and Mediterranean. A total of 564925 V5-V6 reads was obtained. After length filtering, elimination of PCR and random sequencing errors, a raw dataset containing 364633 sequences distributed in 22721 OTUs (97% cut-off) was obtained. Screening for symbiont-related sequences was made on a reduced dataset including 332621 sequences representing 2641 OTUs. Community composition and comparisons were performed on a dataset containing only abundant sequences, overall 306716 sequences in 658 OTUs. On average, dataset sizes were reduced by 35, 41, and 46% of total read numbers and contained 7012, 6397, and 5898 sequences per sample (2 replicate samples per CHEMECOLI). Details on sequence abundances in each sample and dataset, and the mean number of reads per sample are summarized in Table S1.

### Taxonomic richness and diversity

Rarefaction curves based on a similarity threshold of 97% were generated for each substrate type separately (Figure S1). Generally, alfalfa samples showed more diverse (up to 1.6-fold) bacterial communities than their wood equivalents. This was confirmed by bacterial community richness and diversity indices (Table 2). Among alfalfa samples, all long-term ones (>289 days of deployment) from Gulf of Cadiz cold seeps (GoC), Eastern Mediterranean cold seep (EM) and Lucky Strike (L-S) hydrothermal vent on Mid Atlantic Ridge (MAR) (GoC-A-I to GoC-A-IV, EM-A-I and MAR-A-III) presented more diverse bacterial communities than other samples (Table 2). In samples mentioned above the Chao1 index was higher than 1500 and the Shannon index had a value higher than 4.5 compared to wood samples. On any given set of CHEMECOLIs, the bacterial community was less diverse in wood than alfalfa samples (Table 2). Within wood samples GoC-W-II, GoC-W-III and MAR-W-III had the highest values of all considered diversity indices (Table 2). Replicate samples for both alfalfa and wood displayed comparable values for all analyzed diversity indices (Table 2), with two exceptions, namely the replicates in a GoC alfalfa sample (GoC-A-II-1 and GoC-A-II-2), and a short-term wood replicates from MAR (MAR-W-IV-1 and MAR-W-IV-2) (Table 3). Although Shannon and Invsimpson diversity indices showed similar values for the two GoC-A-II replicates, replicates from MAR-W-IV displayed a 6-fold difference in their Invsimpson index (Table 2).

**Table 2:** **Species richness and diversity indices calculated for each sample in Mothur (Schloss et al., 2009) for sequences with OTU identity threshold at 97%**.

**Threshold**		**Index**			
**0.03**		**Richness**		**Diversity**	
**Sample**	**Sobs**	**Chao1**	**ACE**	**Shannon**	**Invsimpson**
**nb_seq**	**4543**				
Contr-A	279.11	526.96	733.44	3.39	11.18
GoC-A-I-1	1196.32	2784.75	4440.27	5.64	52.92
GoC-A-I-2	899.13	2002.70	3446.10	4.99	28.71
GoC-A-II-1	589.27	851.89	912.04	5.22	85.76
GoC-A-II-2	1678.54	4108.65	7117.76	6.39	158.41
GoC-A-III-1	740.86	1558.90	2554.81	4.90	40.70
GoC-A-III-2	1108.94	2297.72	3608.17	5.30	32.70
GoC-A-IV-1	1265.55	2957.10	5319.83	5.71	65.85
GoC-A-IV-2	1396.16	3102.94	4999.33	5.90	73.61
EM-A-I-1	916.56	1984.98	3157.16	5.23	45.95
EM-A-I-2	1058.51	2370.38	3736.46	5.22	35.52
MAR-A-I-1	154.85	207.59	234.98	3.02	9.74
MAR-A-I-2	216.49	421.47	617.40	2.69	6.24
MAR-A-II-1	644.48	1238.09	1883.15	4.42	21.70
MAR-A-II-2	460.93	969.89	1496.23	4.02	18.40
MAR-A-III-1	1041.82	2283.52	3766.65	5.20	29.86
MAR-A-III-2	858.28	2079.23	3740.74	4.41	11.43
HMMV-A-I-1	598.65	1149.13	1601.53	4.37	21.37
HMMV-A-I-2	499.62	862.40	1135.98	4.15	18.58
HMMV-A-II-1	625.12	1315.23	2082.32	4.49	25.48
HMMV-A-II-2	673.01	1283.04	1924.22	4.62	31.34
MAR-A-IV-1	46.91	126.08	192.93	0.81	1.42
MAR-A-IV-2	46.19	102.66	174.43	0.98	1.60
MAR-A-V-1	276.45	604.08	830.39	3.43	11.72
MAR-A-V-2	279.00	508.53	852.31	3.43	11.92
MAR-A-V-C	237.16	503.26	763.51	3.15	9.49
GoC-W-I-1	282.77	433.37	535.60	4.11	27.14
GoC-W-I-2	379.09	656.86	900.68	3.84	12.01
GoC-W-II-1	782.17	1722.28	2878.57	4.61	19.39
GoC-W-II-2	709.60	1440.33	2247.49	4.39	18.23
GoC-W-III-1	529.85	1069.75	1658.48	4.53	30.35
GoC-W-III-2	536.81	1123.29	1637.86	4.71	39.90
GoC-W-IV-1	391.71	714.70	1096.37	4.48	42.43
GoC-W-IV-2	282.05	460.62	510.23	3.67	12.68
EM-W-I-1	367.52	636.47	824.42	4.40	39.49
EM-W-I-2	408.57	741.91	1013.12	4.48	36.14
MAR-W-I-1	178.22	308.73	435.47	2.75	5.51
MAR-W-I-2	97.21	152.68	168.77	2.28	4.06
MAR-W-II-1	178.81	264.22	308.09	3.62	20.69
MAR-W-II-2	257.03	439.33	542.02	3.26	8.00
MAR-W-III-1	691.66	1391.93	2370.47	4.60	28.79
MAR-W-III-2	580.78	1105.29	1658.46	4.43	25.42
HMMV-W-I-1	291.77	568.01	833.71	3.54	13.81
HMMV-W-I-2	407.70	794.03	1054.50	4.12	20.23
HMMV-W-II-1	347.54	625.72	947.47	3.59	10.12
HMMV-W-II-2	434.71	795.56	1031.26	3.99	13.96
MAR-W-IV-1	77.80	106.13	109.30	1.48	2.54
MAR-W-IV-2	354.33	687.95	995.05	3.65	14.37
EM-W-II-1	154.90	266.93	321.80	2.97	10.54
MAR-W-V-1	139.41	291.52	569.19	2.60	7.75
MAR-W-V-2	116.56	238.70	383.18	2.43	6.92
MAR-W-V-C	201.96	442.01	695.70	2.67	6.64

*Sample IDs are described in Table 1*.

**Table 3:** **Symbionts and animal hosts occurrence in geographical regions analyzed in this study**.

**Host family**	**Region**					**Total nb seqs**	**Symbiont occurrence**				**Host occurrence**				**References**
	**C-**	**GoC**	**EM**	**MAR**	**HMMV**		**GoC**	**EM**	**MAR**	**HMMV**	**GoC**	**EM**	**MAR**	**HMMV**	
Alvinocarididae	0	11	13	9669	0	9693	±	±	+	−	−	−	+	−	WoRMS Website
Mytilidae	0	2778	174	4766	1728	9446	+	+	+	+	+	+	+	−	Duperron et al., 2013
Teredinidae	0	1378	14	0	0	1392	+	±	−	−	−	−	−	−	Borges et al., 2014
Tubificidae	0	542	34	10	3	589	+	+	±	±	−	−	−	−	Ruehland and Dubilier, 2010
Siboglinidae	0	393	2	55	37	487	+	±	+	+	+	+	−	+	The World Polychaeta Database Website
Thyasiridae	0	5	0	0	0	5	±	−	−	−	+	+	−	−	Rodrigues and Duperron, 2011
Total nb seqs	0	5107	237	14500	1768	21612									

*Symbiont occurrence is a synthesis of data from Table S5, host occurrence is based on available literature study. Sample IDs are described in Table 1*.

### Taxonomic composition of bacterial communities

**Figure 2:**
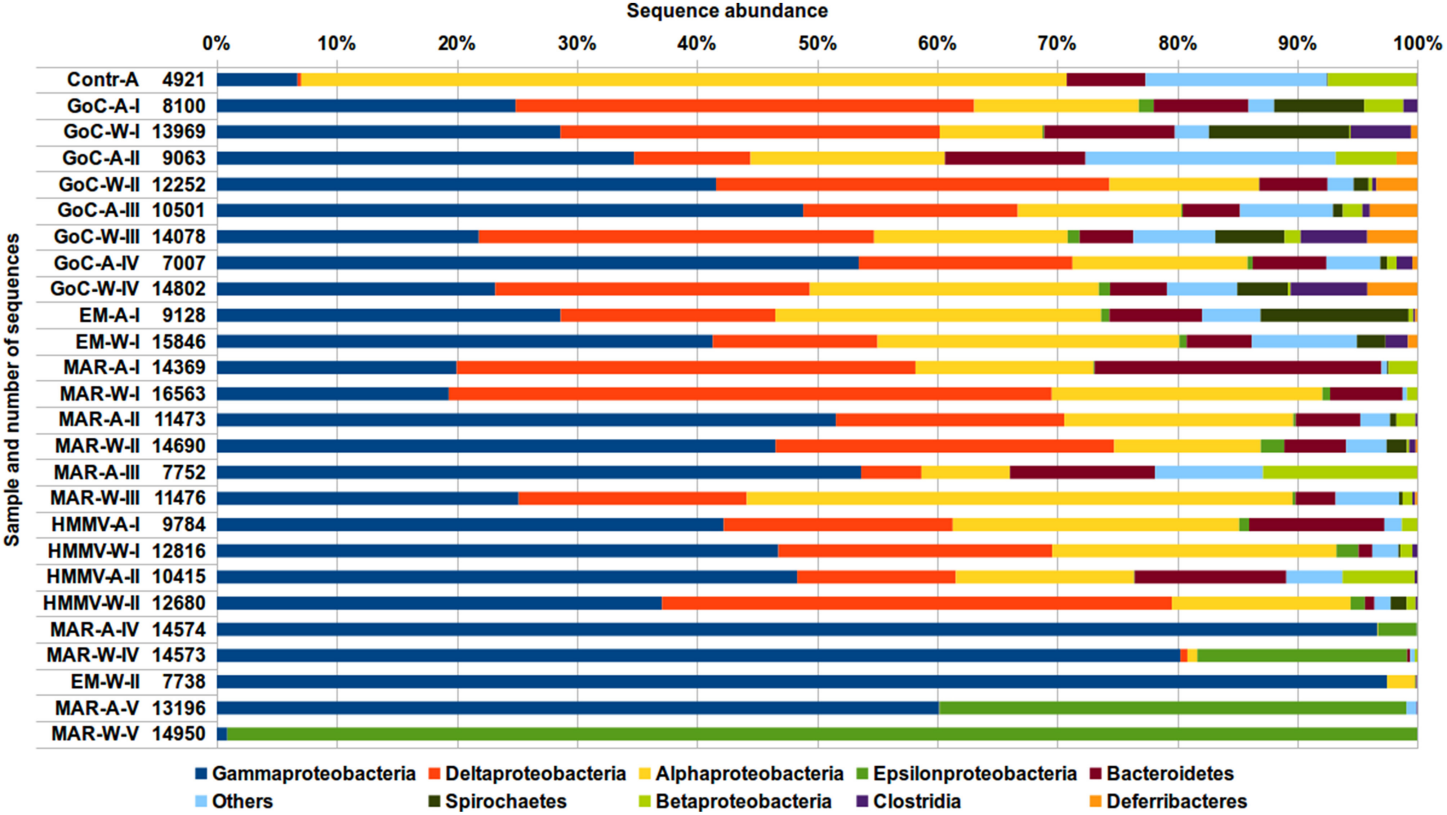
**Composition of bacterial communities at the division or sub-division level based the on V5-V6 region of 16S rDNA gene sequences (OTU identity threshold at 97%)**. Please refer to Table 1 for sample IDs. Abundances are indicated as percentage of total sequences in each sample (numbers close to sample ID's).

At the division or sub-division level, 10 taxa included altogether more than 96% of all sequences present in Dataset_3 (Figure 2; Table S2). Proteobacteria was the most abundant division in this dataset and alone represented above 85% of the total number of reads (Figure 2; Table S2). Within them three taxa (Gamma-, Delta-, and Alphaproteobacteria) corresponded to 77% of the total number of amplicons (Table S2). Gammaproteobacteria were remarkably over-represented in short-term samples (MAR-A/W-IV; EM-W-II and MAR-A-V, Figure 2; Table S2). Epsilonproteobacteria were also highly abundant in short-term samples (MAR-A/W-IV and MAR-A/W-V), while not exceeding 2% of total sequence numbers in long term samples. They were particularly dominant (98%) in the short-term wood sample from M-G on MAR. While almost absent from short-term samples, Delta- and Alphaproteobacteria represented a significant fraction of reads in all long-term samples. Other taxa represented below 30% of reads per sample.

**Figure 3:**
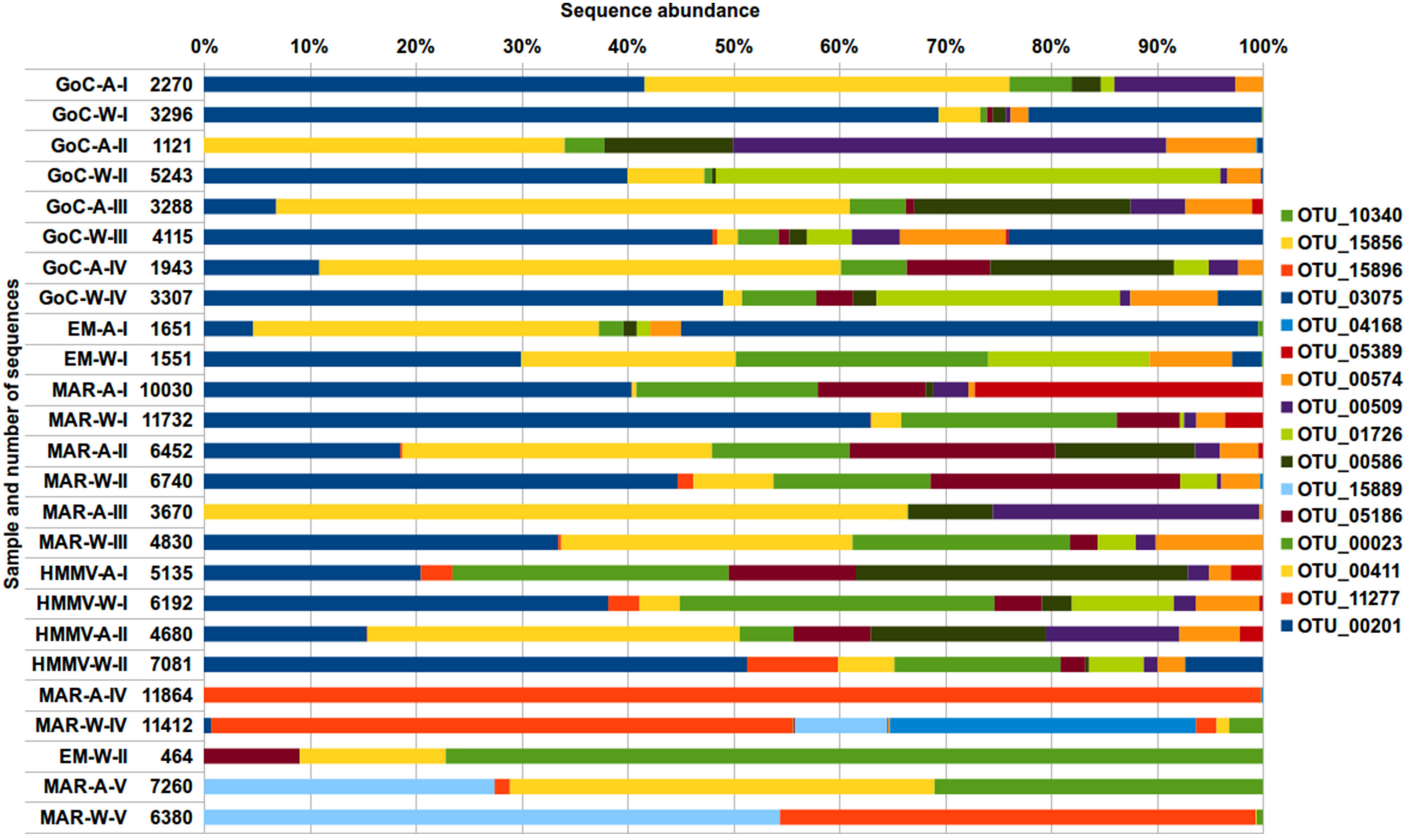
**Percentage of pyrosequencing reads representing the most abundant OTUs (more than 1% of the total number of sequences, i.e., >3000 reads) and assigned to different bacterial phylotypes**. Taxonomic classification was based on comparison of sequences within the SILVA database with OTU identity threshold at 97%. More details on taxonomic assignment of OTUs analyzed is provided in Table S3. Refer to Table 1 for sample ID.

For further community analyses at the OTU level, we focused on the most abundant OTUs, which represented above 1% of the total number of sequences in the dataset (>3000 amplicons; Table S3). 15 OTUs within the Proteobacteria and 1 within the Bacteroidetes matched this criterion, representing 131712 sequences (almost 43% of the total sequences) (Figure 3; Table S3). All were related to marine bacteria. Four major OTUs (OTU_00201, OTU_11277, OTU_00411 and OTU_00023) were abundant in almost all long-term samples and constituted altogether 38 to 86% of the total number of sequences in each sample (Table S3). Communities from short-term experiments were different from the long-term ones, comprising 6 highly abundant OTUs that were rare to absent in long-term deployments (Figure 3; Table S3).

### Comparison among bacterial communities

**Figure 4:**
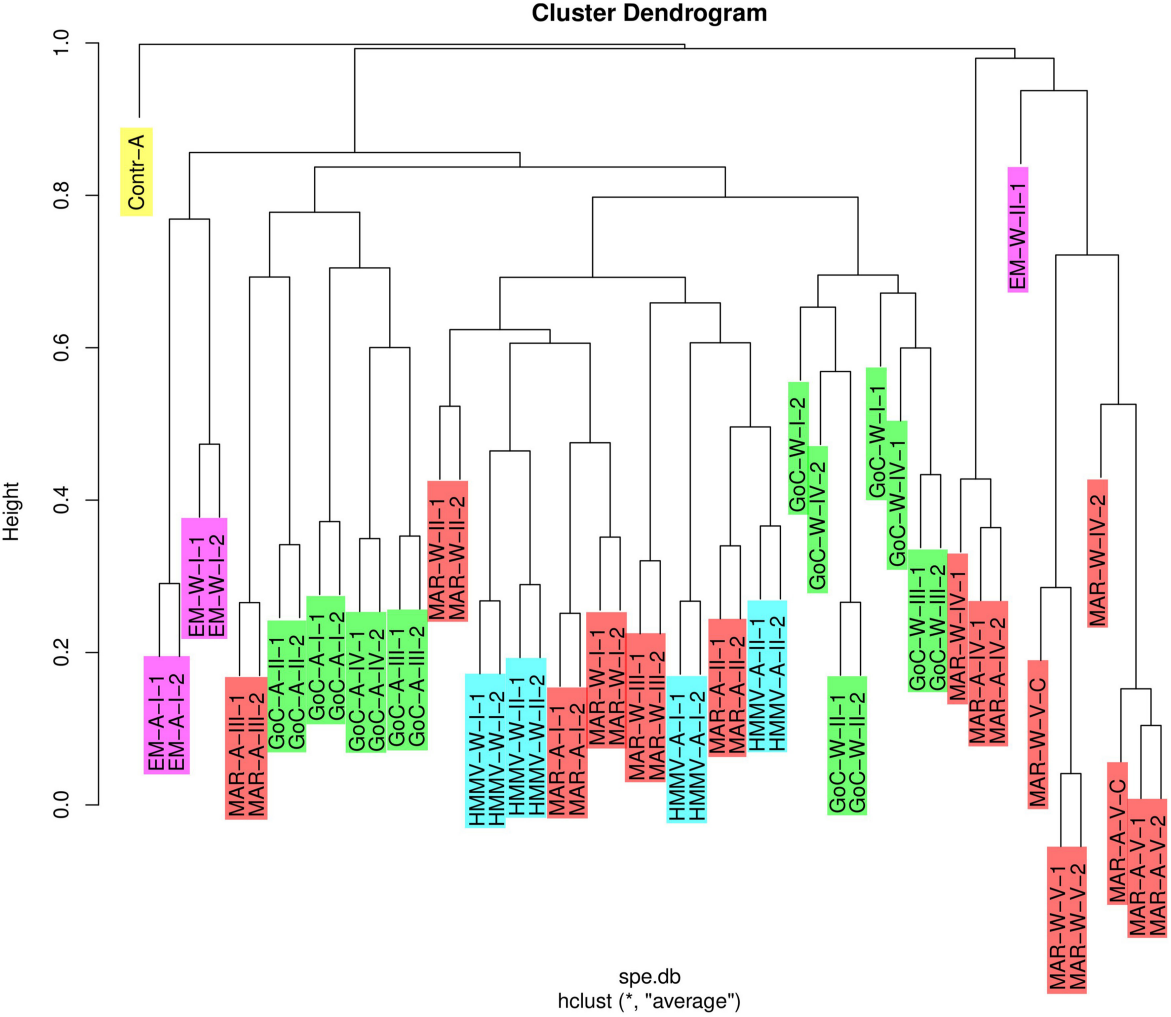
**Dendrogram for average agglomerative clustering generated in R (R Development Core Team, 2013) using Unweighted Pair-Group Method with arithmetic Average (UPGMA) on a Bray-Curtis distance matrix (“hclust()” function in stats package)**. The dendrogram is rooted on the negative alfalfa control (Contr-A-I). Sample IDs are described in Table 1. Color code is the same as that used in Figure 1.

Out of the 24 paired replicates of samples, 21 were grouped together in the UPGMA-based cluster dendrogram based on the Bray-Curtis dissimilarity matrix (Figure 4). The exceptions were two GoC samples (GoC-W-I and GoC-W-IV) and one short-term MAR sample (MAR-W-IV), for which replicates were not the closest relatives. This indicates congruency within most replicates, except for three out of 24 samples. Samples that had been fixed following two different procedures (ethanol fixation for MAR-A/W-V-1/2 and deep freezing for MAR-A/W-V-C) were also on the same branches, indicating that the type of fixation had limited influence on the results. Some groups could be easily distinguished. Both substrates for long-term EM samples (EM-A/W-I) clustered together. Alfalfa substrates from GoC (GoC-A-I to GoC-A-IV) and L-S (MAR-A-III) grouped together; as did wood substrates from GoC (GoC-W-I to GoC-W-IV). A larger group included samples corresponding to both substrates from MAR and HMMV samples (MAR-A/W-I to MAR-A/W-III, HMMV-A/W-I, and HMMV-A/W-II). Finally, all short-term samples formed a clearly distant group (MAR-A/W-IV, MAR-A/W-V, and EM-A/W-II). Interestingly, CHEMECOLIS deployed for different periods of time in the long-term experiment clustered within the same larger groups but do not with each other. These include the GoC Mercator deployments for 289 (GoC-A/W-I) and 731 days (GoC-A/W-II), the MAR Rainbow deployments for 328 and 414 days (MAR-A/W-I and MAR-A/W-II), and the HMMV deployments for 388 and 752 days (HMMV-A/W-I and HMMV-A/W-II). Similar groupings were found on the NMDS graph (Figure S2).

**Figure 5:**
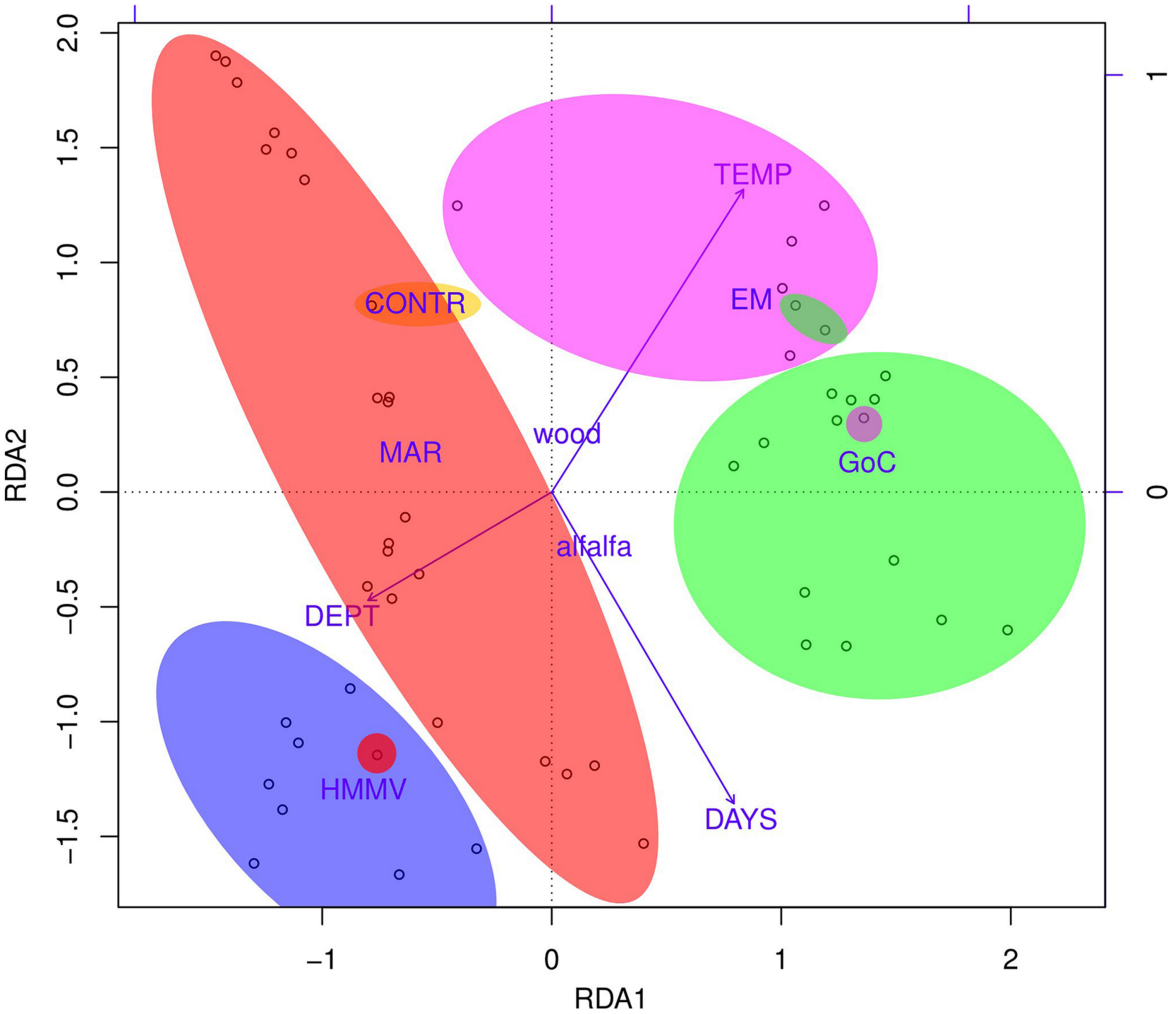
**RDA biplot of the Hellinger-transformed OTU abundance data constrained by all environmental variables, scaling 3**. Substrate type [factor(SUBS)] and region [factor(REG)] were used as factors and water temperature (TEMP), depth (DEPT), and deployment time (DAYS) were used as variables into the constrained RDA, generated in R using “rda()” and “plot()” functions in “vegan” package. Geographic areas are indicated by ellipses with color corresponding to region of origin. Color code follows that of Figures 1, 4. Sample IDs of individual points can be found in Figure S4 and environmental variables and factors are described in Table 1. The influence of each variable and factor and their significance were assessed using ANOVA permutation tests—details in Table S4.

The influence of factors (substrate type, deployment area) and variables (water temperature, duration of deployment and depth) on the community composition was evaluated by constrained RDA (Figure 5 and Figure S4). On the two dimensional graph, clear segregation of samples by region was visible, samples clustering around the centroids representing their region of origin. The influence of the type of substrate was less marked, as centroids representing pine wood and alfalfa were not very distant from one another (Figure 5 and Figure S4). Variables were represented as three vectors with their lengths proportional to the variance explained. Samples from MAR were for example aligned parallel to the vector “DAYS,” according to the duration of deployment: short-term deployments were in the upper left corner of the graph (MAR-A/W-IV and MAR-A/W-V), 1-year in the center left (MAR-A/W-I and MAR-A/W-II), and longest deployments (MAR-A/W-III, 1112 days) in the center bottom (Figure S4). The RDA overall explained 38% of the total variance. The deployment region accounted for 21% of the variance; days, substrate, and depth between 5 to 4%; and the water temperature was responsible for less than 4% of the total variance (Table S4). The ANOVA test confirmed that all factors and variables were statistically significant, based on permutation tests (*p* < 0.001, 99 permutations; Table S4).

### Identification and distribution of bacteria related to chemosynthetic symbionts

A total of 30607 reads within 34 OTUs (~9.2 % of the Dataset_2) have been identified as potential symbionts (>97% of identity with sequences in local database of symbionts) while 21612 reads, distributed in 16 OTUs, had the “symbiont” keyword in at least one of their ten best blast hit definitions (~6.5 % of the Dataset_2). Putatively related symbionts were distributed within 9 potential metazoan host families. Above 22000 belonged to relatives of symbionts associated with *Rimicaris* shrimps, *Idas* mussels and *Lyrodus* shipworms. A phylogenetic tree was computed using representative sequences of potential symbiont OTU, one best blast hit from the local database of symbionts and four best blast hits from public databases (Figure S3). Only the OTUs displaying the keyword “symbiont” within at least one out of ten best blast hits (public database) and above 97% sequence identity with a symbiont sequence were retained as potential candidates for free-living forms of symbionts (Table S5). We obtained 21612 sequences representing 16 OTUs related to symbionts (Table S5). OTUs related to *Rimicaris*–associated Epsilonproteobacteria (97.7–99% identity) represented 9693 reads, followed by *Idas*-associated Symbiont G and Bacteroidetes (6888 and 2558 reads with above 97.4 and 99% identities, respectively), and *Lyrodus* symbionts-related OTUs (1392, above 97.4% identity). Symbionts of *Tubificoides benedii* (589 sequences with 99.7% of identity), relatives of bacteria associated with Siboglinidae polychaetes (487, 97.6–99.7% identity) and Thyasiridae bivalves (5 sequences only with 97.9% identity) were far less abundant (Table S5).

The *Rimicaris* symbiont-related OTUs were highly abundant in short-term deployments in MAR, very rare in EM and GoC, and absent everywhere else. Relatives of *Idas* Bacteroidetes and Symbiont G, Siboglinidae symbionts and those of Tubificidae were ubiquitous (GoC, EM, MAR, HMMV). Those of *Lyrodus* were present only in GoC and EM, and those of Thyasiridae bivalves only in GoC. The occurrence of symbiont-related OTUs was related to the host presence in the corresponding regions (Table 3). Shrimps and their symbionts co-occured on the MAR (WoRMS Website^1^,) with only few symbiont related sequences in GoC and EM. Thyasirids (Rodrigues and Duperron, 2011) and their symbionts co-occurred in GoC, while symbionts were absent in MAR, HMMV and EM. Symbionts of *Idas* mussels and siboglinids were present in all regions, while the hosts were absent at HMMV (Duperron et al., 2013) and MAR (World Polychaeta Database Website^2^) respectively. Symbionts of *Tubificoides benedii* were also ubiquitous while those of teredinids could be detected mainly in GoC and EM. Metazoans of these two families occur in shallow water.

## Discussion

### Methodology

All CHEMECOLI samples were analyzed in duplicates. Estimated diversity and richness indices of most duplicates were comparable, save for two exceptions in which Chao1 and ACE indices varied 5–9-fold between replicates for a long-term sample from GoC and for short-term one from MAR. These two exceptions emphasize the need for sample replication. The UPGMA dendrograms evidenced the very short distances between most replicated samples. Similar groupings were observed on the RDA (Figure 5) graph, in which replicates were most often in very close vicinity to each other. Finally, high similarity was observed between samples stored in 96% ethanol and deep frozen at −80°C. Ethanol treatment is thus an appropriate method for sample storage and conservation in view of the difficult conditions experienced sometimes on board oceanographic cruises and during shipment.

### Bacterial colonization of pine wood and alfalfa substrates in deep-sea chemosynthesis-based ecosystems

A variety of devices have been designed to study bacterial colonization in deep-sea ecosystems such as hydrothermal vents, in most cases using relatively inert surfaces (examples in Reysenbach et al., 2000; López-García et al., 2003; Rassa et al., 2009). Wood substrates have been introduced more recently (Fagervold et al., 2012, 2013; Bienhold et al., 2013). In our study, bacterial communities colonizing alfalfa were usually more diverse than those colonizing wood samples (Figure S1; Table 2). This is not unexpected because wood displays high content of lignin, which consists in refractory hydrophobic polymers harder to degrade than cellulose, and because alfalfa offers greater surface per volume for bacterial colonization. The overall OTU diversity in short-term deployments was lower than in samples deployed for more than 289 days. After 289 days, no correlation was observed between community richness and deployment duration (data not shown), suggesting that diversity may have reached saturation sometime in between 14 and 289 days. Short-term samples were usually recovered during distinct legs of the same cruise, while long-terms samples were recovered during follow-up cruises, which occurred on a yearly basis, explaining the lack of intermediate deployment times. These would however be crucial to understand how fast saturation is reached.

The bacterial community in long-term samples included only 10 bacterial subdivisions (Figure 2; Table S2). Three of them (Alpha-, Delta-, and Gammaproteobacteria) were over-represented if compared to others, as shown before (Fagervold et al., 2012, 2013; Bienhold et al., 2013). Among the most dominant OTUs (Figure 3; Table S3) were members of the Oceanospirillales and Alteromonadales, facultative anaerobe growing on cellobiose and previously found on sunken wood (Fagervold et al., 2012). Within the Deltaproteobacteria, OTUs related to Mycococcales and Desulfobacteriales, and two groups of SRBs detected by Bienhold et al. (2013) (OTU refs in Bienhold et al., 2013: Deltaproteobacteria_03,_24,_27,_50,_55,_283,_421, and _737), were identified. Finally, Bacteroidetes were found, which are often chemoorganotrophs specialized in degrading various biopolymers such as cellulose (Kirchman, 2002). The composition of short-term communities was different, and except for the wood sample from M-G, was dominated by Gammaproteobacteria. This is comparable with the 1-day deployment control sample in Bienhold's study (2013). Epsilonproteobacteria were over-represented in the short-term M-G wood sample thanks to the high abundance of *Rimicaris exoculata* ectosymbiont (Table S3), which is discussed below. The abundance of Epsilonproteobacteria is consistent with their dominance at seep and vent ecosystems and their documented quick colonization of newly formed habitats including wood (Reysenbach et al., 2000; López-García et al., 2003; Huber et al., 2010; Fagervold et al., 2012, 2013; Bienhold et al., 2013).

### Major factors influencing the microbial community composition

The RDA explains less than 40% of the total variance observed in the community composition. It means that other factors and variables, likely including environmental parameters not measured during sampling, play a major role. These are hard to obtain because only time-series acquired in the immediate vicinity of CHEMECOLIs and over the course of the experiment would make sense. Nevertheless, physico-chemical data could explain another significant fraction of the total variance (Lee et al., 2014). Among factors considered here, the deployment area contributed 21% of the variance. This is evident from the UPGMA, NMDS and RDA graphs which cluster samples according to their area of origin, with few exceptions (i.e., L-S sample MAR-A/W-III). EM samples are grouped and GoC alfalfa and wood samples build two rather distinct groups. Interestingly, MAR and HMMV samples appear more mixed. This suggests that communities from vent MAR and cold seep HMMV deployments are less distinct, despite the great geographical distance and very different latitudes. This observation reminds of a previous study in which deep ocean microbial communities from poles and low latitudes differed less than corresponding samples from the euphotic surface waters (Ghiglione et al., 2012). Other factors and variables each contribute 5% or less to the variance. The fact that substrate does not explain much variance is probably due to the similar composition of alfalfa and wood, both being plant-derived material. The consequence is that although alfalfa communities are more diverse, the most abundant OTUs tend to be shared. Alfalfa may host a greater number of rare OTUs. More caution is needed when looking at other variables. Duration of deployment for example only explains 5% of the total variance, yet short-term samples are remarkably less diverse and cluster very far from long-term samples in the various analyses. This apparent lack of congruency between the results of RDA and comparisons is clearly due to a sampling bias. Short-term samples (10–13 days) represented only 5 of the 25 deployed CHEMECOLIs, the other being deployed for 289–1112 days. Their quantitative contribution to the variance in the overall datatset is thus low, and only if we had as many short terms as long terms samples would we have a correct estimate of the percentage of variance explained by deployment duration. Long-term CHEMECOLIS deployed at the same site for different durations do not display the most similar communities, but their communities do not cluster very far from one another, suggesting a certain level of stability at this stage. If we had better coverage of intermediate times and a more balanced design, duration of deployment could become a major explanatory factor (Palacios et al., 2009; Fagervold et al., 2012). Although the temperature variable is well equilibrated with 7 sites displaying typical deep-sea temperatures (2–4.6°C) against six others ranging from 10 to 13°C, it explains less than 4% of the total variance. Depths cover a broad bathymetric range (354 to 2300 m) and samples are distributed rather homogeneously, yet it explains only 4% of the variance. Depth is usually not considered a major factor in the aphotic zone of the ocean (Fagervold et al., 2013; Zhu et al., 2013). Neither latitude nor water temperature showed significant correlations with estimated microbial diversities in the previous large scale study by Ghiglione et al. (2012). Overall, the geographical area of deployment seems to be the most important factor influencing the microbial colonization in this study. Short term samples are different from long terms in both their overall diversity as well as community composition. Both substrates share similar dominant bacteria and alfalfa displays greater diversity thanks to rare OTUs. Communities become saturated in term of both diversity and composition between 14 and 289 days after deployment. For longer periods, bacterial composition appears to be relatively stable, as samples from the same sites group together. Only marine microbes colonize both substrates in CHEMECOLIs, no typical contaminants could be detected. The bacterial taxonomic composition identified in colonization devices compared to other studies (Fagervold et al., 2012, 2013; Bienhold et al., 2013) confirms that the degradation of organic matter starts to take place within the first 2 weeks after immersion.

### Colonization of substrates by symbiont-related bacteria

The sequence dataset was screened for sequences related to chemosynthetic symbionts of metazoans. Based on percentage similarity (97%) criterion with documented symbionts, we retained sequences closely related to documented symbiotic bacteria as reasonably supported candidates. Despite that the short length of reads hampers the quality of phylogenetic reconstructions, the combination of sequence similarity and phylogenetic relatedness appears a rather conservative approach. Almost 70% of symbiont-related sequences were found on pine wood (Table S1). The higher diversity within alfalfa samples may indeed “dilute” symbiont-related sequences in higher numbers of other bacterial OTUs, making their discovery less likely. Putative free-living forms of symbionts or symbiont-related bacteria from five host metazoan families were identified. The most abundants were related and 99% identical to Epsilonproteobacteria ectosymbionts of *Rimicaris exoculata*. In this species, bacteria quickly re-colonize the shrimp's gill chamber after each molt, estimated to occur every 10 days (Zbinden et al., 2004; Corbari et al., 2008). Our results are in good agreement with this constraint. Shrimp symbiont-related OTUs were indeed the most abundant in the short-term MAR samples (MAR-A/W-IV and MAR-A/W-V, 10–14 days), and absent from all long-term deployments. They thus seem to be pioneer and efficient colonizers of available surfaces at vents. This questions the specificity of the symbiotic association. It may well be that bacteria behave as opportunists colonizing the new cuticle when it appears, as they would colonize any surface. The molt cycle would favor iterative colonization by the same bacteria simply by renewing the surface available every few weeks.

Relatives of gill endosymbionts of the mytilid *Idas* sp. were also abundant, mostly the Symbiont-G group and the Bacteroidetes, two symbiont groups which were recently discovered in species from the eastern Mediterranean and the Gulf of Cadiz (Duperron et al., 2008; Rodrigues et al., 2013). The usual sulfur-oxidizers are much rarer, though a few were found. Finding free-living relatives of mussel symbionts was not unexpected, given that environmental acquisition of symbionts is supported in *Idas modiolaeformis* based on the absence of bacteria within gonads and gametes (Gaudron et al., 2012). In a recent study, Laming et al. (2014) showed that environmental symbiont acquisition took place at the early juvenile stage, after settlement.

The occurrence of *Lyrodus* and *Osedax* symbionts-related OTUs with identities between 97.4 and almost 100% was more unexpected in our samples, as shipworms live in shallow coastal waters (Borges et al., 2014) and *Osedax* is a bone-eating specialist (Rouse et al., 2004; Glover et al., 2005). *Lyrodus* gill endosymbionts have been proposed to produce cellulolytic enzymes that contribute to the host's ability to digest wood (Waterbury et al., 1983), and multiple bacterial phylotypes can co-occur in its gill bacteriocytes (Distel et al., 2002). Although the *Lyrodus pedicellatus* symbiont transmission mode remains still unknown, vertical transmission has been evidenced for its relative Bankia setacea (Sipe et al., 2000). But others wood-eating bivalves (*Xylophaga* spp. and *Xyloredo* spp.) were abundant in most samples and although we carefully avoided processing any animal tissue in our analyses, we cannot rule out that some bacterial symbionts were released during processing. Knowing that Bienhold et al. (2013) suggested a close phylogenetic relationship between symbionts of the shallow and deep-sea wood-boring bivalves we cannot objectively conclude whether the identified OTUs are actually free-living forms of *Lyrodus pedicellatus* or *Xylophaga* spp. symbionts. Presence of an OTU related to *Tubificoides benedii* symbionts is also surprising as these oligochaete worms are found in eutrophic coastal sediments, but at the same time their ectosymbionts have been shown to belong to clades that consist almost exclusively of bacteria associated with invertebrates from deep-sea hydrothermal vents (Ruehland and Dubilier, 2010). The co-occurrence of thyasirid bivalves and their symbionts, represented in our study by only 5 sequences in GoC, may be explained by the mode of life of these animals burrowing deeply in sediments (Dufour and Felbeck, 2003).

### Symbiont transmission and colonization

All symbiont-related OTUs corresponded to environmentally-transmitted bacteria. The existence of free-living forms is thus expected, although their natural habitat is not documented (Gros et al., 1996; Harmer et al., 2008). Symbiont-related sequences were quite frequent in a few samples, and some were present on several sites. The abundance of symbionts on short-term wood samples indicates that they are probably efficient colonizers, although little is known about the mechanism of symbiont acquisition from the environment. The *Idas* sp. symbiont-G related-OTUs were on the other hand found in moderate numbers, but in almost all samples. Most of symbionts detected shared the same distribution pattern as their hosts (Table 3). An exception was the shipworms' symbiont in GoC and in EM, while their host occurs only in coastal shallow waters (Borges et al., 2014), but this may be because of close relatedness with symbionts of *Xylophaga* spp. which are present. We also did not expect the occurrence of Siboglinidae polychaetes symbionts on MAR, nor those of *Idas* sp. in HMMV. The detection of unexpected symbiont-related phylotypes (Table 3; Table S5) fuels the debate on the connectivity between different deep-sea sites (Vrijenhoek, 2010). Moreover, it confirms that short- and long-term colonization devices and high-throughput sequencing are good tools to further investigate the habitat and densities of free-living relatives of the symbionts.

The next step will be to distinguish between free-living close relatives of symbionts and true free-living forms of symbionts, but this will be tricky. Symbiont-specific fluorescence *in situ* hybridization (FISH) probes may not be able to properly discriminate, and low abundances of free living relatives may render difficult their detection and quantification on substrata, which show a high level of background autofluorescence (data not shown). Finally, the analyzed hypervariable region of 16S rRNA seems to be too short to be a good target for highly specific probes. Standardized devices are currently being deployed in other geographical regions and in other habitats. There is a high potential to improve the protocol we applied in our study with the increasing length of pyrosequencing reads, thus making the identification of microbes more reliable. For better understanding of the influence of environment on the microbial communities, the chemistry of water should be monitored precisely in the proximity of our colonization devices.

### Conflict of interest statement

The authors declare that the research was conducted in the absence of any commercial or financial relationships that could be construed as a potential conflict of interest.

## Footnotes

^1^WoRMS, World Register of Marine Species. http://www.marinespecies.org/index.php

^2^The World Polychaeta Database http://www.marinespecies.org/polychaeta/

## Supplementary material

Figure S1 **Rarefaction analysis for the alfalfa grass (A) and pine wood (W) samples**. The curves were generated for 97% levels of OTU using Mothur (Schloss et al., 2009). Sample IDs are described in Table 1.

Figure S2 **NMDS biplot of a Bray–Curtis dissimilarity matrix of the OTU abundance data generated in R (R Development Core Team, 2013) using “metaMDS()” and “plot()” functions in MASS package**. Sample data has been added using weighted averages. The short-term samples and the negative control are far from the long-term samples (green rectangle), the latter forming two cloud-like groups. Along the horizontal axis (NMDS1), long-term samples are roughly separated by substrate type, with alfalfa on the left and wood on the right (blue ellipse). The vertical axis displays a clear separation between samples from GoC and EM (red ellipse) and samples from MAR and HMMV (upper part), with the alfalfa sample from L-S (MAR-A-III) in between. Sample IDs are described in Table 1.

Figure S3 **Phylogenetic tree based on sequences of V5-V6 hypervariable region of 16S rDNA gene obtained from the “potential symbiont” dataset and on respective sequences of known symbionts in public database**. Phylogenetic relationships among sequences were estimated with MEGA6 (Tamura et al., 2013) from a 250-bp ClustalX alignment (Larkin et al., 2007) using distance methods and neighbor-joining. Bootstrap values were computed on 1000 replicates. Each OTU is represented by sample ID followed by representative OTU number and total number of sequences in the respective OTU. Reference sequences from public database are represented by the accession number followed by the strain name. Based on the tree, OTUs related to environmental sequences were considered as doubtful candidates were not used for further analysis.

Figure S4 **RDA biplot of the Hellinger-transformed OTU abundance data constrained by all environmental variables, scaling 3**. The figure is the same as Figure 5 but samples IDs are detailed.

Table S1 **Detailed distribution of sequencing data in datasets used in this study**. Refer to Table 1 for sample IDs.

Table S2 **The total number of V5-V6 sequences at OTU identity threshold of 97% in each bacterial division or sub-division and relative percentage in total effective bacterial sequences per sample**. Refer to Table 1 for sample ID.

Table S3 **The total number of V5-V6 sequences at OTU identity threshold of 97% in the most abundant OTUs, represented by more than 1% of the total number of sequences in the dataset (>3000 amplicons)**. OTUs were assigned to different bacterial phylotypes by Blast on SILVA 115 database with OTU identity threshold at 97%. The relative percentage was normalized by the total number of the most abundant bacterial sequences per site. Refer to Table 1 for sample ID.

Table S4 **Estimation of the influence of each variable and factor and their respective significance in redundancy analysis (RDA), assessed using ANOVA permutation tests in R (R Development Core Team, 2013)**. Sample IDs are described in Table 1.

Table S5 **The total number of sequences of symbiont-related OTUs identified by the “symbio” key word in at least one of their four best BLAST hit definitions or high similarity with a sequence from the symbiont database and confirmed by the phylogenetic proximity**. Only OTUs displaying a symbiont sequence as their closest relative on the phylogenetic tree were retained. Sample IDs are described in Table 1.
