# Supplementary figures and images for: Colonization of plant substrates at hydrothermal vents and cold seeps in the northeast Atlantic and Mediterranean and occurrence of symbiont-related bacteria
Source: Front Microbiol. 2015 Feb 27;6:162. doi: 10.3389/fmicb.2015.00162 (PMC4343019; doi:10.3389/fmicb.2015.00162)

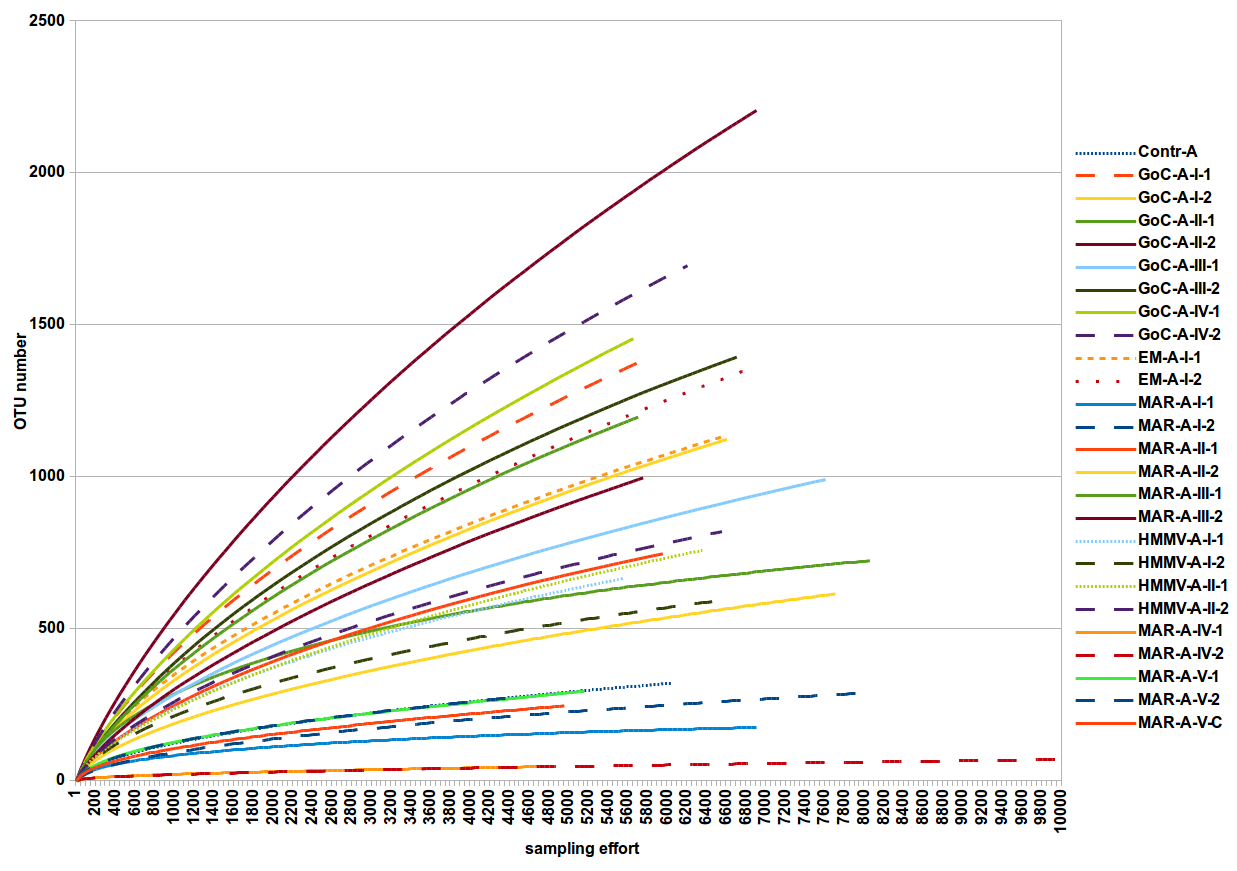

Supplement: Figure S1 — Rarefaction analysis for the alfalfa grass (A) and pine wood (W) samples. The curves were generated for 97% levels of OTU using Mothur (Schloss et al., 2009). Sample IDs are described in Table 1. [file Image1.TIF]

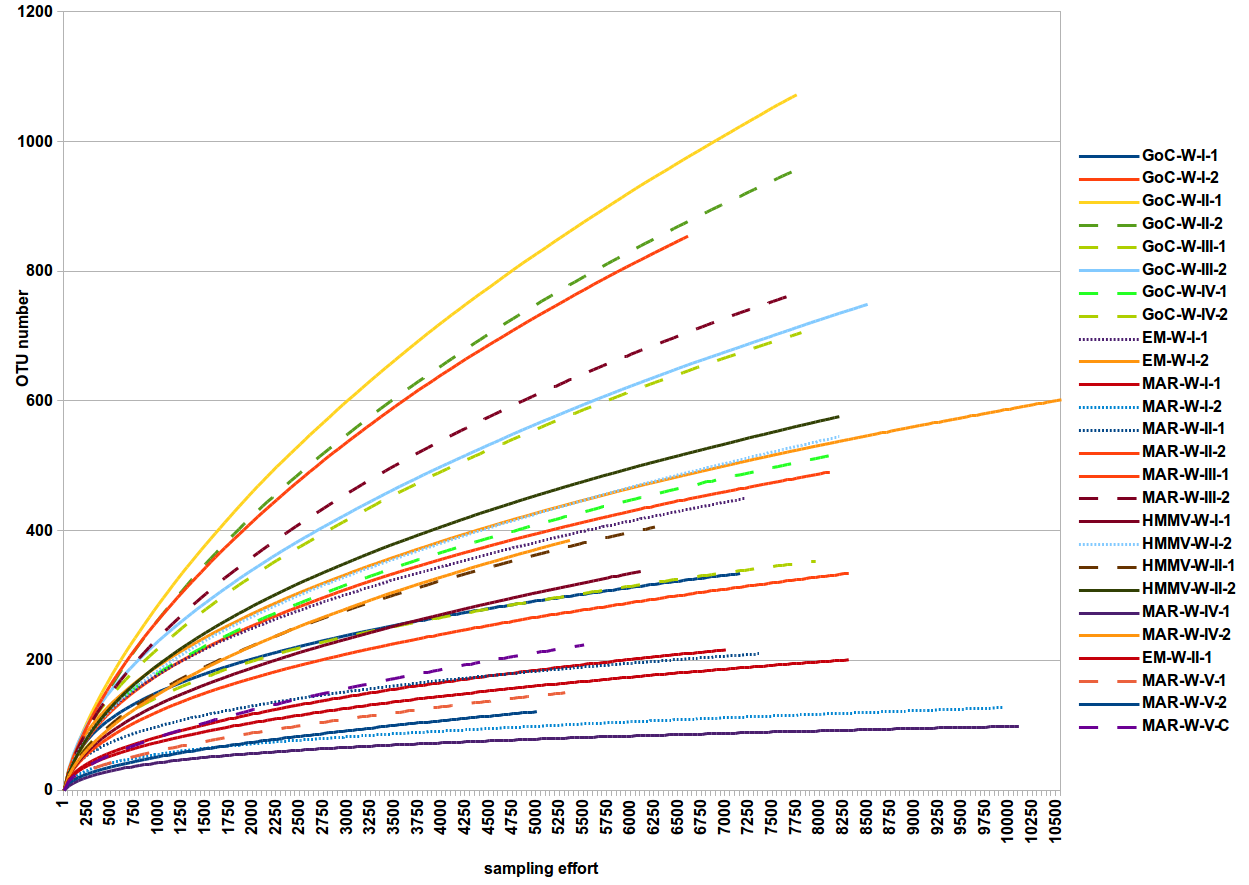

Supplement: Figure S2 — NMDS biplot of a Bray–Curtis dissimilarity matrix of the OTU abundance data generated in R (R Development Core Team, 2013) using “metaMDS()” and “plot()” functions in MASS package. Sample data has been added using weighted averages. The short-term samples and the negative control are far from the long-term samples (green rectangle), the latter forming two cloud-like groups. Along the horizontal axis (NMDS1), long-term samples are roughly separated by substrate type, with alfalfa on the left and wood on the right (blue ellipse). The vertical axis displays a clear separation between samples from GoC and EM (red ellipse) and samples from MAR and HMMV (upper part), with the alfalfa sample from L-S (MAR-A-III) in between. Sample IDs are described in Table 1. [file Image2.TIF]

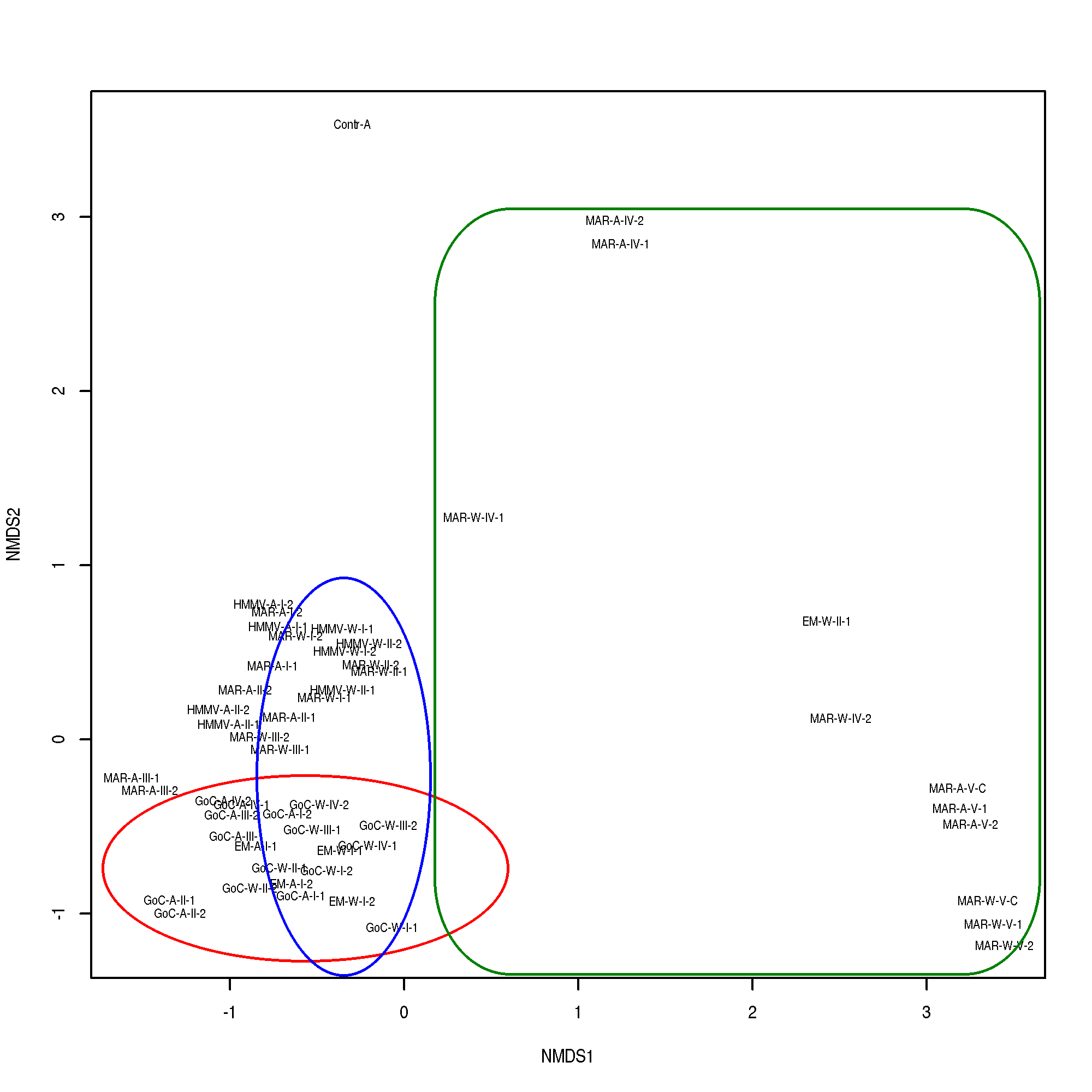

Supplement: Figure S3 — Phylogenetic tree based on sequences of V5-V6 hypervariable region of 16S rDNA gene obtained from the “potential symbiont” dataset and on respective sequences of known symbionts in public database. Phylogenetic relationships among sequences were estimated with MEGA6 (Tamura et al., 2013) from a 250-bp ClustalX alignment (Larkin et al., 2007) using distance methods and neighbor-joining. Bootstrap values were computed on 1000 replicates. Each OTU is represented by sample ID followed by representative OTU number and total number of sequences in the respective OTU. Reference sequences from public database are represented by the accession number followed by the strain name. Based on the tree, OTUs related to environmental sequences were considered as doubtful candidates were not used for further analysis. [file Image3.TIFF]

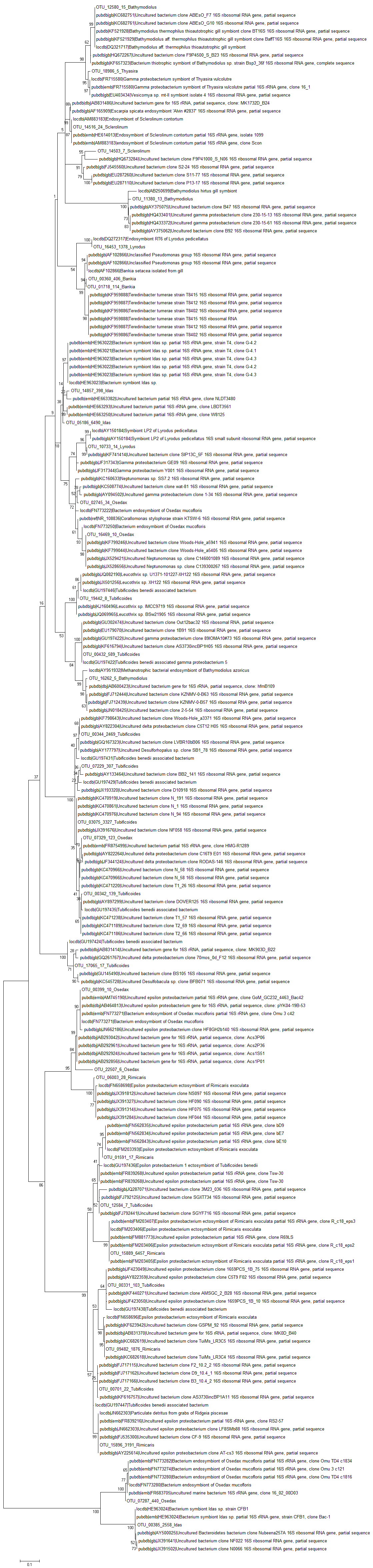

Supplement: Figure S4 — RDA biplot of the Hellinger-transformed OTU abundance data constrained by all environmental variables, scaling 3. The figure is the same as Figure 5 but samples IDs are detailed. [file Image4.TIF]

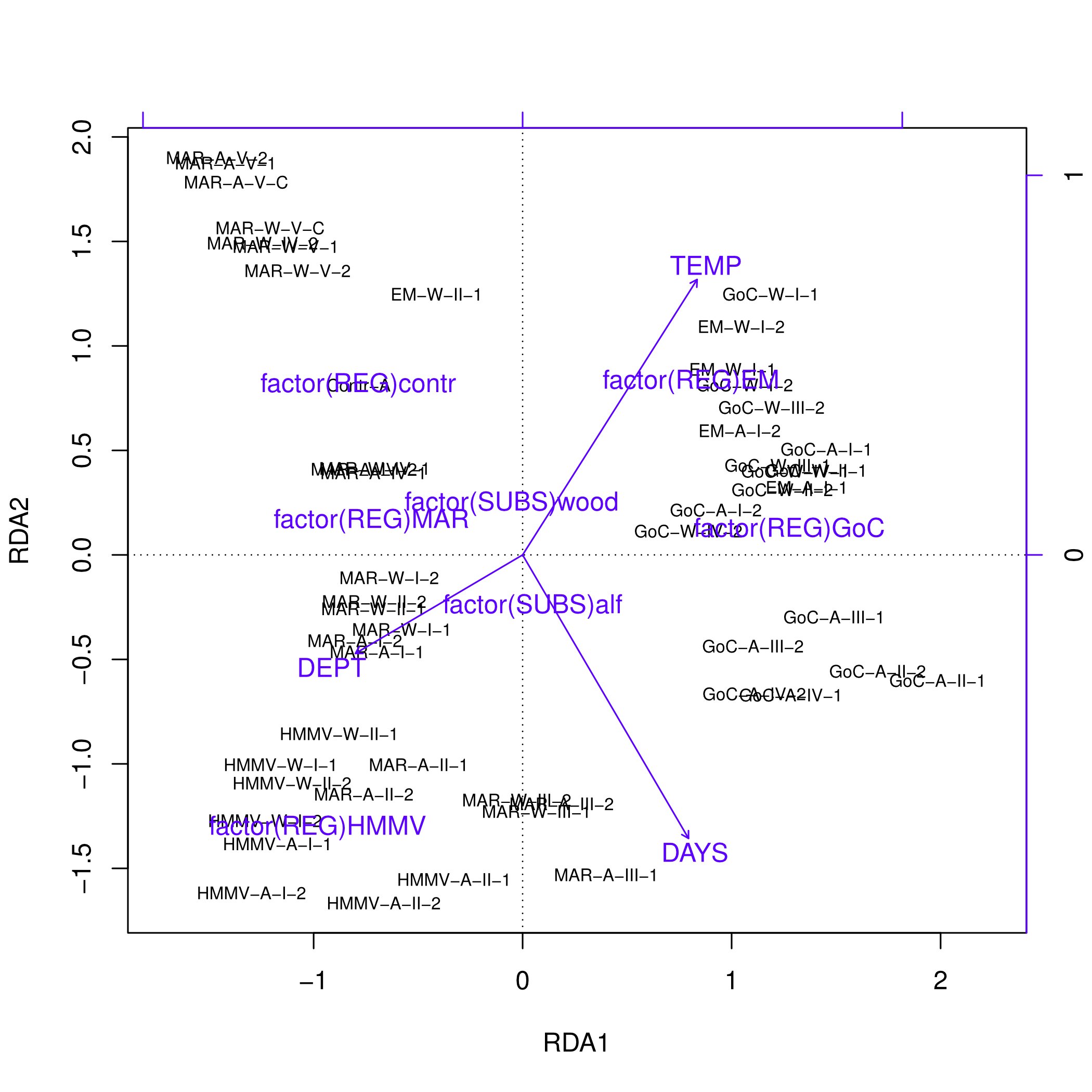

Supplement: Supplementary file 11 [file Image5.JPEG]
